# Cross-sectional study of COVID-19 infection in patients with rheumatic diseases in a sample of a Damascene population, Syria

**DOI:** 10.1097/MS9.0000000000000274

**Published:** 2023-03-14

**Authors:** Naram Khalayli, Maysoun Kudsi

**Affiliations:** Damascus University, Damascus, Syrian Arab Republic

**Keywords:** COVID-19, inflammatory markers, musculoskeletal, pneumonia, rheumatic diseases

## Abstract

Coronavirus infections have been responsible since 2019 for respiratory illnesses with varying severity worldwide. Worst outcomes from coronavirus (COVID-19) have been reported in older patients andthose with comorbidities like rheumatic diseases. Some drugs used for treating rheumatic diseases are used in patients with COVID-19. Based on the limited data, rheumatic diseases do not seem to affect the disease course of COVID-19. We aimed to analyze the course of COVID-19 infections in patients with rheumatic diseases.

**Methods:**

A self-reported questionnaire was distributed online and to patients admitted with respiratory involvement. Data included demographic information, clinical presentation, severity, comorbidities, and laboratory parameters. Cases were matched by age, sex, the month of admission, and COVID-19 respiratory injury for patients with rheumatic diseases and patients without rheumatic diseases.

**Results:**

Twenty-two patients (4.4%) had rheumatic diseases before the COVID-19 infection. There were no differences in the use of treatment for COVID-19 infections in previous or present therapy or comorbidities. We found no significant difference in the duration of COVID-19 symptoms before admission, duration of hospital stay, or chest Xray Brixia score between the two groups. The lymphocyte count was lower in the patient group, while lactate dehydrogenase, ferritin, and D-dimer concentrations were higher compared to the control group. Thrombotic events were similar in rate.

**Conclusion:**

The poorer outcome from COVID-19 infections in patients with rheumatic diseases is related to older age and the presence of comorbidities rather than the rheumatic disease type or its treatment.

## Introduction

Highlights

- Cross-sectional study of 22 patients with coronavirus disease 2019 (COVID-19) and rheumatic diseases and a control group of patients with COVID-19 but without a rheumatic disease.
- Cases were matched by age, sex, the month of admission, and COVID-19 respiratory injury.
- Found no significant difference in the duration of COVID-19 symptoms before admission, duration of hospital stay, or chest X-ray Brixia score between admitted patients with rheumatic disease and COVID-19 infection and the control group.
- Concluded that poorer outcome from COVID-19 infections in patients with rheumatic diseases is related to older age and the presence of comorbidities rather than the rheumatic disease type or its treatment.

Coronavirus infections have been responsible since 2019 for respiratory illnesses with varying severity worldwide^1^. It is important to assess its clinical characteristics in patients with rheumatic diseases. Poorer outcomes from COVID-19 have been reported in older patients and those with comorbidities. As they had lower immunity status due to the disease itself or its treatment, developing serious infections that should be considered^2^.

Some of the drugs used for treating rheumatic diseases, like hydroxychloroquine, interleukin-6 inhibitors, and interleukin-1 antagonists, are being used in patients with COVID-19, especially in the presence of cytokine storm syndrome^3^.

No data about COVID-19 infection among patients with rheumatic diseases have been published; most research is in the form of small case series and reports^3^–^8^.

Based on the limited data, rheumatic diseases do not seem to affect the disease course of COVID-19. We aimed to analyze the course of COVID-19 infection in patients with rheumatic diseases.

## Patients and methods

A cross-sectional/case–control study was conducted in the Rheumatology departments of several University Hospitals from October 2019 to June 2021. Ethical approval was obtained from the Ethics Committee of the Faculty of Medicine, Damascus University, Syria, and this study was done according to the principles of the Declaration of Helsinki.

Patients signed a written informed consent form upon hospital admission. This paper has been reported in line with the STROCSS (Strengthening the Reporting of Cohort Studies in Surgery) criteria^9^.

An online questionnaire was created and distributed in addition to admitted patients with respiratory involvement and rheumatic diseases. Data included demographic information, clinical presentation, duration of the symptoms, contact with a general practitioner, laboratory parameters, immunological findings, severity, comorbidities, concomitant disease, ongoing therapy management of background medications, measurement of infection prevention, quarantine measures, presence of other infected family members or friends, and rheumatic disease types and manifestations.

Matched by age, sex, and time of admission for COVID-19 respiratory injury cases, the control group was enrolled.

Fever, fatigue, nonproductive cough, dyspnea, runny nose, myalgia, arthralgia, nausea, vomiting, diarrhea, headache, and anosmia, are clinically referred to as a COVID-19 infection^10^.

The diagnosis of a COVID-19 infection was confirmed by a positive PCR assay from nasopharyngeal swabs and/or the presence of interstitial pneumonia on chest computerized tomography.

According to the WHO guidelines^11^, patients were classified into severe cases if they needed respiratory support and mild disease in the absence of severity criteria.

The severity of pneumonia in COVID-19 patients was defined by the Brescia-COVID Respiratory Severity Scale (BCRSS): a score of 3 or greater means that a patient needs respiratory help (high-flow oxygen, biphasic or continuous positive airway pressure)^10^,^12^.

Brixia score was used for the evaluation of interstitial pneumonia. The overall score was between 0 and 18, divided into a score of 0 for no lung abnormalities; a score of 1 for interstitial infiltrates; a score of 2 for interstitial and alveolar infiltrates, with a predominance of interstitial infiltrates; and a score of 3 for interstitial and alveolar infiltrates with a predominance of alveolar infiltrates^13^.

All patients admitted with confirmed COVID-19 pneumonia and rheumatic diseases and controls with confirmed COVID-19 pneumonia but without rheumatic diseases were matched by age, sex, and month of hospital admission.

Cases and controls were analyzed for differences in the disease course, radiological findings, presence of inflammatory laboratory measurements, and treatments.

The inflammatory markers were defined as a lymphocyte count less than l000 cells/ml, and two of the following three criteria: D-dimers more than 1000 ng/ml, serum ferritin more than 500 ng/ml, and lactate dehydrogenase more than 300 U/l.

### Statistical analysis

Categorical variables were reported as proportions or percentages, whereas continuous variables were reported as median and interquartile values. Quantitative variables were compared using the Mann–Whitney test, whereas categorical variables were compared using Fisher’s test. *P* values less than 0.05 were considered significant.

## Results

Twenty-two patients (4.4%) had a rheumatic disease before the COVID-19 infection. There were 14 (63.63%) patients with confirmed COVID-19 and 8 (36.36%) patients with a clinical picture suggestive of the infection. The median age was 64 years old in the confirmed COVID-19 infection group and 55 years old in the suspected clinical infection group, with 17 females and 5 males. Confirmed cases of COVID-19 were more frequently observed in female patients than in male patients [11 (78.57%) vs. 3 (21.43%)]. Meanwhile the females also outweighed the males in the remaining eight patients with a clinical picture suggestive of the infection [6 (75%) vs. 2 (25%)]. All patients stopped their therapies following the onset of COVID-19 symptoms.

The rheumatic diseases were: 4 systemic lupus erythematosus patients; 10 rheumatoid arthritis patients; 2 Sjogren’s syndrome patients; 2 Bechet’s disease patients; 1 scleroderma patient; and 1 vasculitis patient.

Confirmed cases of COVID-19 were older than suspected cases (64 vs. 55 years), with comorbidities like obesity, arterial hypertension, diabetes, cardiovascular disease, and chronic renal failure, respectively. There were no differences in the use of types of treatments between confirmed and suspected cases of COVID-19 infection. Therapy consisted of steroids, then disease-modifying anti-rheumatic drugs, and finally biologics.

COVID-19 admitted cases of pneumonia occurred in 11 (78.75%) of confirmed cases (Table 1).

**Table 1:** Clinical features of 22 patients with rheumatic and musculoskeletal diseases and coronavirus disease 2019 (COVID-19) infections.

Characteristics	Patients with confirmed COVID-19 (*n*=14), *N* (%)	Patients with a clinical picture suggestive of COVID-19 (*n*=8), *N* (%)
Age (years)	64 (53–76)	55 (47–64)
Number of patients >65 years of age	8 (57.14)	2 (25)
Sex		
Female	11 (78.57)	6 (75)
Male	3 (21.43)	2 (25)
Rheumatic diseases		
Rheumatoid arthritis	7 (50)	3 (37.5)
Systemic lupus erythematosus	4 (28.57)	0 (0)
Systemic sclerosis	1 (7.14)	0 (0)
Sjogren’s syndrome	2 (14.28)	0 (0)
Vasculitis	0	1 (12.5)
Bechet disease	0	2 (25)
Treatment		
Glucocorticoids	9 (64.28)	4 (50)
Colchicine	0 (0)	2 (25)
DMARDs		15 (29)
Hydroxychloroquine	7 (50)	4 (50)
Methotrexate	8 (57.14)	3 (37.5)
Leflunomide	1 (7.14)	0 (0)
Azathioprine	3 (21.42)	2 (25)
Biologics		
Infliximab	1 (7.14)	0 (0)
Golimumab	3 (21.42)	1 (12.5)
Rituximab	1 (7.14)	0 (0)
Comorbidities		
Hypertension	7 (50)	4 (50)
Cardiovascular disease	3 (21.42)	2 (25)
Obesity	9 (64.28)	5 (62.5)
Diabetes	4 (28.57)	2 (25)
Chronic renal insufficiency	1 (7.14)	0 (0)
Hospital admission for COVID-19 pneumonia	11 (78.57)	0 (0)
Deaths	2 (14.28)	0 (0)
Close contact	3 (21.42)	4 (50)

DMARDs, disease-modifying anti-rheumatic drugs.

Patients admitted to the hospital were older than those who were not, with a median age of 70 years (60–76) vs. 54 years (47–74). We found no differences in previous or present therapy or comorbidities. Two patients with rheumatic and confirmed COVID-19 infection died (Table 2).

**Table 2:** Clinical features of two deceased patients with confirmed coronavirus disease 2019 infection.

	Sex	Age	Rheumatic disease	Disease therapy	Comorbidities
Patient (1)	Female	74 years	Rheumatoid arthritis	Oral prednisone, 10 mg/day Golimumab	Obesity, hypertension, diabetes
Patient (2)	Female	37 years	Systemic lupus erythematosus	Oral prednisone, 15 mg/day Mycophile Mycophenolate One dose of rituximab	Cardiovascular disease, hypertension, chronic renal failure

We compared 11 patients with a rheumatic disease requiring admission to the hospital for COVID-19, with 21 other patients without rheumatic disease as the control group, admitted to our hospital matched in age and sex.

We found no significant differences in the duration of COVID-19 symptoms before admission, duration of hospital stay, or chest X-ray Brixia score.

The lymphocyte count was lower in the patient group, while lactate dehydrogenase, ferritin, and D-dimer concentrations were higher compared to the control group.

A thrombotic event during hospital admission occurred in three cases (27.27%) and six controls (28.77%). Among the three cases in the patient group with rheumatic diseases, two cases were venous thrombosis, and one was pulmonary embolism. Moreover, four patients with venous thrombosis, one pulmonary embolism, and one thrombotic cerebrovascular accident in patients of the control group.

During hospitalization, intravenous dexamethasone was started in all patients (11 of 11 patients), in addition to treating 4/11 patients with tocilizumab for worsening respiratory conditions: 2 patients with rheumatoid arthritis, who were previously treated by golimumab (anti-tumor necrosis factor) and 2 patients with lupus, one of whom was treated with rituximab and the other with azathioprine and hydroxychloroquine.

There were no differences in the duration of hospital admission, chest X-ray Brixia score, or therapies administered during hospital admission between cases who had high inflammatory markers and the patients with low/no inflammatory measurements (Table 3).

**Table 3:** Comparison of data cases and controls.

Characteristics	Control	Patients
Age	71 (58–77)	69 (53–76)
Sex		
Female	12	6
Male	11	5
Duration of symptoms before hospital admission (days)	7 (5–10)	8 (7–10)
Duration of hospital stay (days)	14 (9–20)	15 (11.5–27.5)
Chest X-ray Brixia score at admission	7 (4–11)	7 (4–10)
Chest X-ray Brixia score at discharge	7 (5–10)	9 (8–11)
Lactate dehydrogenase (>300 U/l)	12/21	6/11
Ferritin level (>500 μg/l)	14/21	8/11
D-dimer (>1000 ng/ml)	5/21	4/11
Lymphopenia (<1000 per mm^3^)	12/21	8/11
Comorbidities		
Hypertension	14	6
Cardiovascular disease	6	3
Obesity	9	7
Diabetes	4	2
Chronic renal insufficiency	0	1
Deaths	4/21	2/11
Treatment		
Antiviral drugs	17	7
Hydroxychloroquine	18	11
Intravenous dexamethasone or oral prednisone	19	11
Tocilizumab	6	4
Oxygen	19	9
High-flow oxygen, continuous positive airway pressure, biphasic positive airway pressure	12	6

## Discussion

The impact of severe acute respiratory syndrome coronavirus 2 infection on patients with the chronic rheumatic disease is currently unclear. Data indicates that COVID-19 in patients with rheumatic and musculoskeletal diseases is not rare in a district with high dissemination of COVID-19 infection^1^,^2^, and when compared to patients without rheumatic diseases, patients older than 65 years have a higher rate of comorbidities, and a more severe disease course^2^,^14^, like our results.

Usually, the high inflammatory process, including the cytokine storm, increases the need for respiratory support^3^,^12^ and requires the use of dexamethasone or tocilizumab, or both^12^.

We used dexamethasone and/or tocilizumab based on the severity of respiratory symptoms, per the BCRSS^12^. In cases requiring ventilatory support (BCRSS ≥3), tocilizumab was used after the inefficiency of dexamethasone use^15^, just as we did.

Preinfection use of biologics in patients with rheumatic disease may prevent a more severe disease course^3^,^12^, but because of the small number of our patients with rheumatic disease, we were unable to confirm this knowledge. Although the deceased patients in our cases were previously on biologics, we found no differences in background therapies when comparing confirmed COVID-19 cases with suspected cases and when comparing patients who had COVID-19 pneumonia and higher inflammatory process with those who did not, which means that the prognosis of COVID-19 infection is related to the other risk factors rather than the rheumatic disease itself or its present therapy^16^. In our cases, older age was the main risk factor for hospital admission and death, and this is in concordance with the reports in the American College of Rheumatology Guidelines^17^. Hypertension and obesity in patients with confirmed infections were associated with more severe symptoms of COVID-19 in our study, and this is compatible with other studies^18^,^19^.

The limitations of our cases were the small number of patients infected both by COVID-19 and rheumatic diseases and with each disease alone, which made us unable to analyze and compare the effects of a COVID-19 infection on these patients.

## Conclusion

Previous systemic immune disease and immunosuppressant treatment are not the top risk factors for COVID-19 infection outcomes in people with rheumatic diseases. Older age and other comorbidities were more important than the dose or type of immunosuppressants.

## Ethical approval

Ethical approval was given by the Ethics Committee of the Faculty of Medicine, Damascus University.

## Patient consent

Written informed consent was obtained from the patient for the publication of this research. A copy of the written consent is available for review by the Editor-in-Chief of this journal on request.

## Sources of funding

No funding was obtained for this research.

## Author contribution

N.K.: wrote the ‘Patients and methods’ and the ‘Discussion’; M.K.: wrote the ‘Abstract’ and ‘Introduction’ and participated in the discussion and also did final edits and revisions. Both authors collected the data and analyzed the results.

## Conflicts of interest disclosure

There are no conflicts of interest.

## Research registration unique identifying number (UIN)

1. None.

## Guarantor

Prof Maysoun Kudsi is the guarantor for this particular study.

## Data availability statement

The data supporting the results of this article are included in the article’s references.

## Provenance and peer review

Not commissioned, externally peer-reviewed.
